# Salinomycin-loaded Nanofibers for Glioblastoma Therapy

**DOI:** 10.1038/s41598-018-27733-2

**Published:** 2018-06-20

**Authors:** Mohammad Norouzi, Zahra Abdali, Song Liu, Donald W. Miller

**Affiliations:** 1 Graduate Program of Biomedical Engineering, University of Manitoba, Winnipeg, MB Canada; 2 Department of Biosystems Engineering, Faculty of Agricultural and Food Sciences, University of Manitoba, Winnipeg, MB Canada; 3 Department of Pharmacology and Therapeutics, University of Manitoba, Winnipeg, MB Canada

## Abstract

Salinomycin is an antibiotic that has recently been introduced as a novel and effective anti-cancer drug. In this study, PLGA nanofibers (NFs) containing salinomycin (Sali) were fabricated by electrospinning for the first time. The biodegradable PLGA NFs had stability for approximately 30 days and exhibited a sustained release of the drug for at least a 2-week period. Cytotoxicity of the NFs + Sali was evaluated on human glioblastoma U-251 cells and more than 50% of the treated cells showed apoptosis in 48 h. Moreover, NFs + Sali was effective to induce intracellular reactive oxygen species (ROS) leading to cell apoptosis. Gene expression studies also revealed the capability of the NFs + Sali to upregulate tumor suppressor Rbl1 and Rbl2 as well as Caspase 3 while decreasing Wnt signaling pathway. In general, the results indicated anti-tumor activity of the Sali-loaded NFs suggesting their potential applications as implantable drug delivery systems in the brain upon surgical resection of the tumor.

## Introduction

Glioblastoma multiforme (GB) is the most common malignant and aggressive brain tumor in adults whose median survival time is usually less than 2 years post-diagnosis. Systemic drug delivery to the brain tumor is fairly restricted because of the blood–brain barrier (BBB)^1,2^. While surgical resection and radiation therapy is standard of care for patients with GB, local recurrence of the brain tumor is often observed and responsible for high mortality in this cancer. Therefore, local drug delivery to the tumor vicinity can circumvent the BBB, provide sustained exposure to chemotherapy to inhibit local recurrence of the brain tumor and improve the patient’s survival time. Furthermore, local drug delivery can typically decrease systemic side effects while providing a high concentration of the drugs at the tumor site with less frequent drug administration^1,3,4^.

Several classes of implantable drug delivery systems have been introduced including wafers, gels, rods and films, the majority of those are typically made of biodegradable polymer-based systems avoiding a second surgery for the implant removal^5,6^. For example, chemotherapy through local intracranial drug delivery with biodegradable polymeric wafers (Gliadel^®^) delivering carmustine is currently being utilized as an adjunct to surgery and radiation for GB patients^7^.

In the recent years, nanofibers have attracted much attention as implantable drug delivery systems in the tumor site or at the surgical resection margins owing to their attractive features such as large surface-area-to-volume ratio linked to high loading capacity as well as high encapsulation efficiency^8,9^. Various agents such as doxorubicin (DOX)^5^, paclitaxel (PTX)^10^, cisplatin (CDDP)^11^ for chemotherapy, multiwalled carbon nanotubes (MWCNTs)^12^ and gold nanorods (Au NRs)^13^ for photothermal therapy as well as iron oxide nanoparticles (Fe_3_O_4_ NPs)^14,15^ for hyperthermia therapy, have been incorporated into nanofibers to treat various types of cancers.

Electrospinning is a straightforward and versatile technique to fabricate ultrafine fibers with diameters ranging from nanometer to micrometer from a variety of materials such as polymers, inorganics and hybrid (organic–inorganic) compounds^16–18^. Electrospun nanofibers have been extensively utilized in biomedical, tissue engineering as well as drug delivery applications. Therapeutic agents can be incorporated into electrospun nanofibers through blend, coaxial, and emulsion electrospinning^8^. Generally, drug release from nanofibers follows either diffusion-controlled or degradation-controlled mechanisms depending on the polymer and nanofiber structure^5^.

Salinomycin is an antibacterial and ionophore anticoccidial therapeutic drug, which has recently been introduced as a novel alternative to traditional anti-cancer drugs^19,20^. In 2009, Gupta *et al*.^21^, found salinomycin, a drug which was utilized as an antibiotic in veterinary medicine, to be 100 times more effective than Taxol (paclitaxel) in killing breast cancer stem-like cells. More recent studies have confirmed salinomycin toxicity towards gastrointestinal sarcoma, osteosarcoma, and colorectal cancers^22^. Whilst, the molecular mechanisms of salinomycin toxicity are not fully understood, several studies have reported that intracellular Ca^2+^, cytochrome c, and caspase activation can be involved in salinomycin-induced cytotoxicity^23^. Moreover, salinomycin can target cancer stem cells and inhibit the Wnt/β-catenin pathway that is crucial for stem cell self-renewal^24^. In addition, salinomycin can induce mitophagy, mitoptosis and increased mitochondrial membrane potential while causing strong and time-dependent ATP-depletion in cancer cells^23^.

In this study, salinomycin was incorporated into the nanofibers for the first time through electrospinning to be utilized as an implantable drug delivery system for local glioblastoma therapy and tumor recurrence prevention. Poly (lactic-co-glycolic acid) (PLGA), a Food and Drug Administration (FDA)-approved biocompatible polymer, was employed to fabricate the drug-loaded nanofibers. Results indicates that the NFs + Sali can induce apoptosis in GBM cells and therefore this platform may be further developed as an implantable drug delivery system in the brain cavity after surgical resection of the tumor.

## Results and Discussion

**Figure 1:**
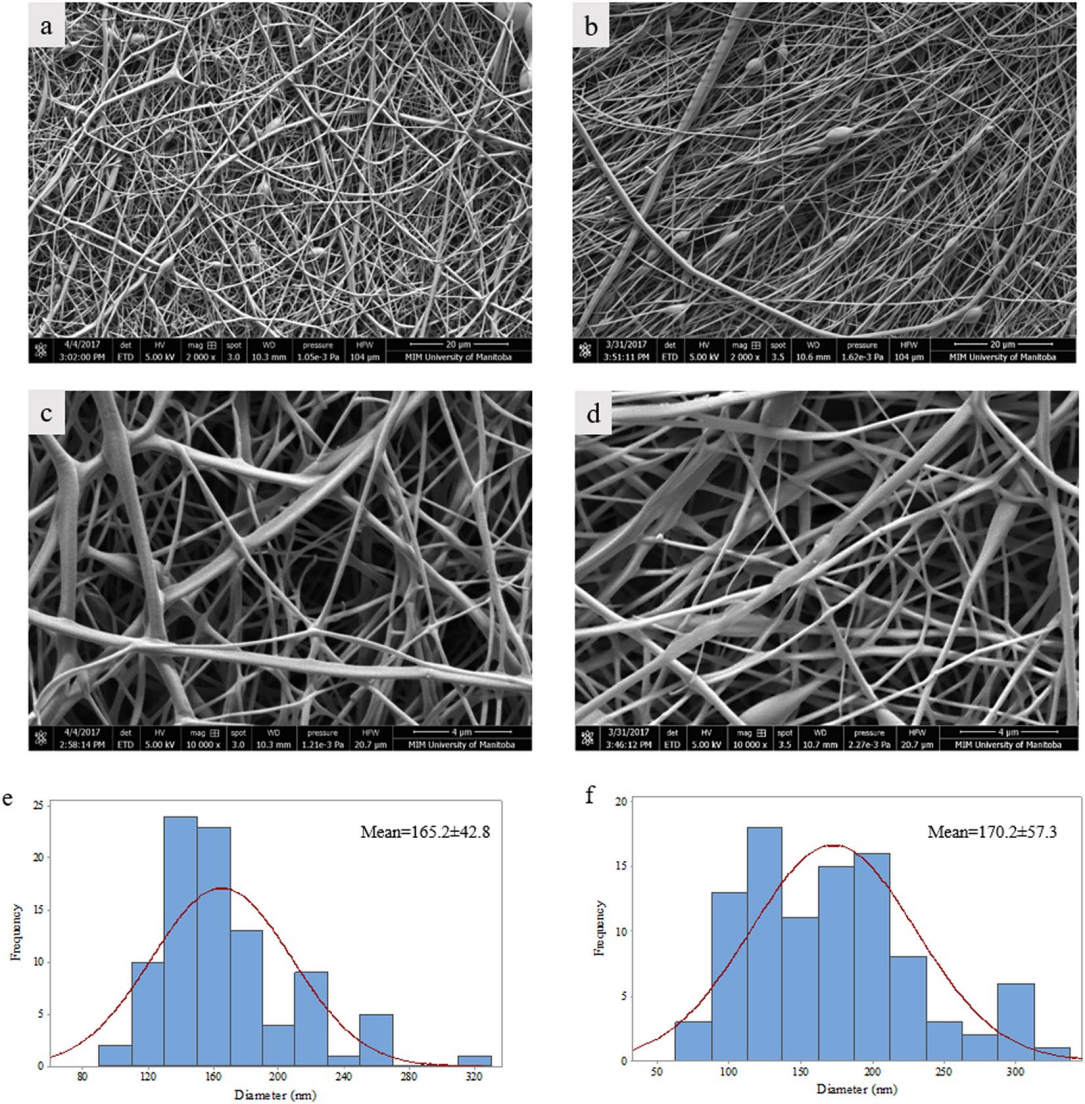
SEM images of Electrospun PLGA NFs (**a**,**c**) and PLGA NFs + Sali (**b**,**d**). Histograms of diameter distribution of PLGA NFs (**e**) and PLGA NFs + Sali (**f**).

PLGA nanofibers containing salinomycin were fabricated through blend electrospinning. Figure 1 shows SEM images of the PLGA NFs (a,c) and PLGA NF + Sali (b,d). Each of the scaffolds were formed of randomly oriented nanofibers with a well-interconnected porosity that can be beneficial in capturing cancer cells preventing migration and metastasis. The average diameter of PLGA NFs and PLGA NFs + Sali was 165 ± 42 nm and 170 ± 57 nm, respectively and the histograms in Fig. 1(e and f) illustrate normal distributions of the nanofiber diameters.

**Figure 2:**
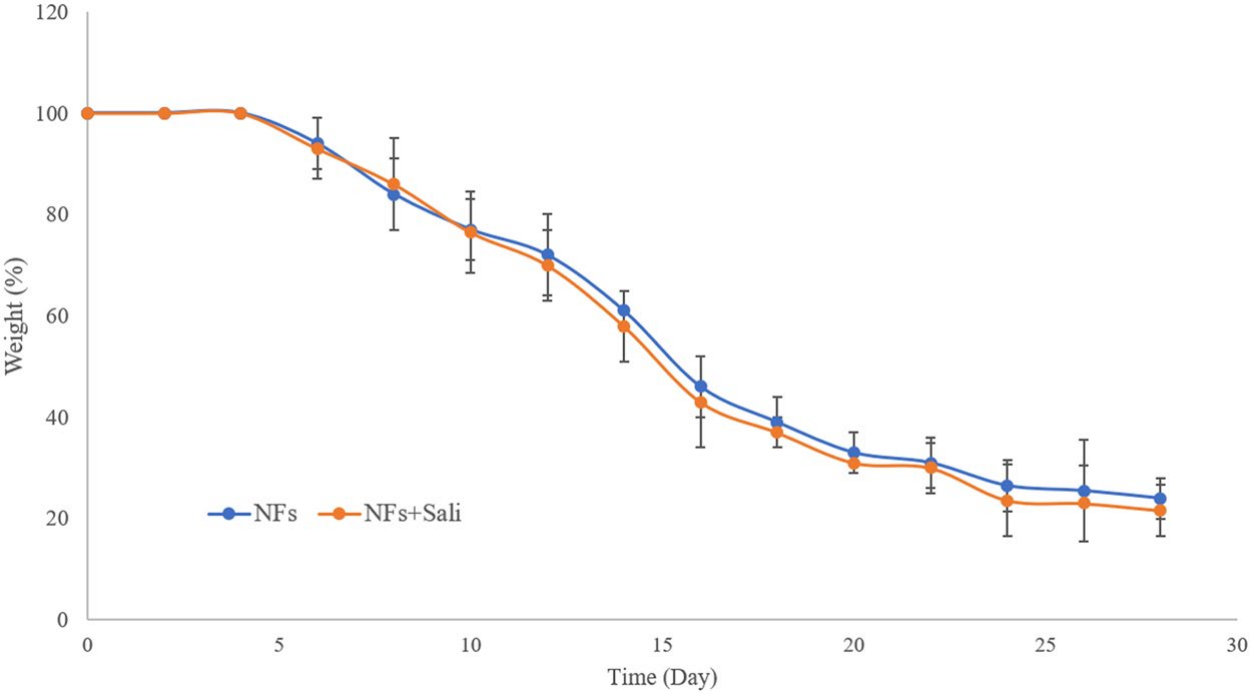
*In vitro* degradation profile of the PLGA NFs and PLGA NFs + Sali as a function of incubation time in PBS (pH 7.4, 37 °C). Data was presented as mean ± S.D, and n = 3.

The *in vitro* degradation profile of the PLGA and PLGA + Sali nanofibers is shown in Fig. 2. No appreciable loss of nanofiber material was observed over the first 4 days. Thereafter, a relatively constant rate of weight loss was observed with the nanofibers and they were mostly degraded within 4 weeks, attributed to the low molecular weight of the PLGA. No significant difference was observed between the degradation pattern of PLGA NFs and PLGA NFs + Sali (Fig. 2). This was expected as the amount of Sali contained within the PLGA NFs represented a small fraction of the overall material weight.

Generally, biodegradable polymers can be categorized as either surface eroding or bulk eroding polymers. Polyesters such as PLGA show mainly bulk degradation, which occurs faster than surface erosion^25^. Degradation rate of PLGA nanofibrous scaffolds is a function of several parameters such as the lactic to glycolic acid ratio, molecular weight as well as the scaffold architecture by which drug release kinetics can be tailored^8,26^. In addition, biodegradability of the implantable drug-loaded nanofibers circumve nts foreign body immune responses and second surgery removal procedures^27^.

**Figure 3:**
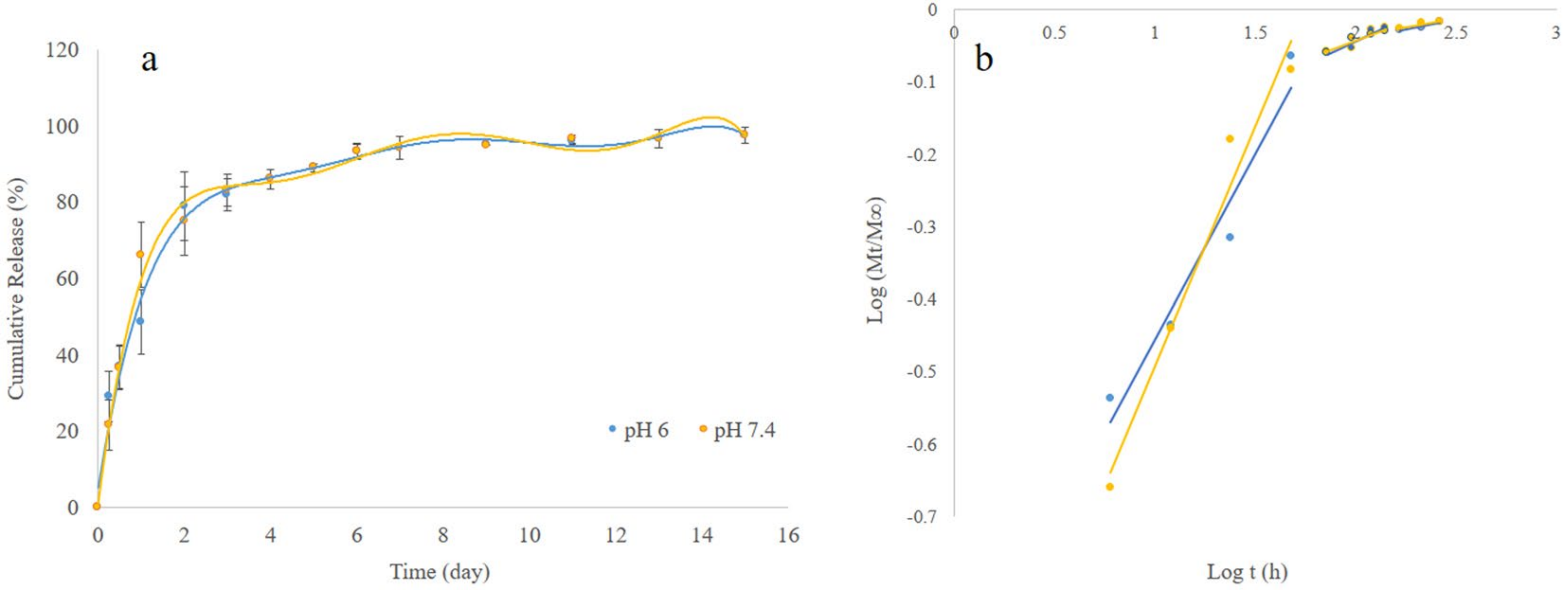
*In vitro* release profile of Sali from PLGA NFs + Sali at pH 7.4 and 6 (**a**); mechanism analysis of Sali release according to Siepmann and Peppas equation (**b**).

The release of salinomycin from the PLGA nanofibers was examined at pH 6 and 7.4 Generally, the release profile consisted of two stages: a rapid release over 4 days in which ca. 80% of the drug was released followed by a slower release over 10 days in which the remaining encapsulated drug was released (Fig. 3). A similar pattern of release has been reported for other drugs from electrospun nanofibers. For instance, Laiva *et al*.^28^, reported 70% release of titanocene dichloride from electrospun polycaprolactone (PCL)/silk fibroin (SF) nanofibers in 2 days, while the released concentration reached the maximum in 6 days. In another work, Sasikala *et al*.^29^, conjugated bortezomib (BTZ) on PLGA nanofibers through mussel-inspired surface functionalization and reported a rapid burst release of 70% in 12 h at pH 6. Similarly, doxorubicin (DOX)-loaded micelles encapsulated in the core of core-shell nanofibers demonstrated ca. 70% release at 4 days followed by a gradual release over time^30^. In addition, there was no difference in Sali release profile at pH 6 compared to pH 7.4. This is important as solid tumor environments can become hypoxic thus changing drug release and drug response^31^.

Given that, there was no significant weight loss of nanofibers within the first four days, this first stage of drug release can be explained by the diffusion of drug located near the surface of the nanofibers. The second stage of Sali release, from the nanofibres was likely attributed to partial polymer degradation^8,32,33^. The Sali release kinetics are in agreement with the degradation profile of the PLGA nanofibers. To confirm that both diffusion and degradation components were involved in Sali release from PLGA nanofibers, results were analyzed based on Siepmann and Peppas equation (Table 1)^34,35^. Using the data from the current study, the exponent n calculated by Siepmann and Peppas equation, shows a value of 0.5, indicating that anomalous transport was the mechanism controlling Sali release over the first 4 days while initiation of polymer bulk degradation can be related to the drug release over the remaining period.

**Table 1:** *n* and *k* values for Sali release at pH 7.4 and 6.

	Time (day)	n	k	R^2^
pH 7.4	0–4	0.5	0.1	0.93
	4–8	0.1	0.74	0.91
	8–12	0.04	0.77	0.99
pH 6	0–4	0.5	0.38	0.95
	4–8	0.1	0.85	0.93
	8–12	0.04	0.89	0.99

**Figure 4:**
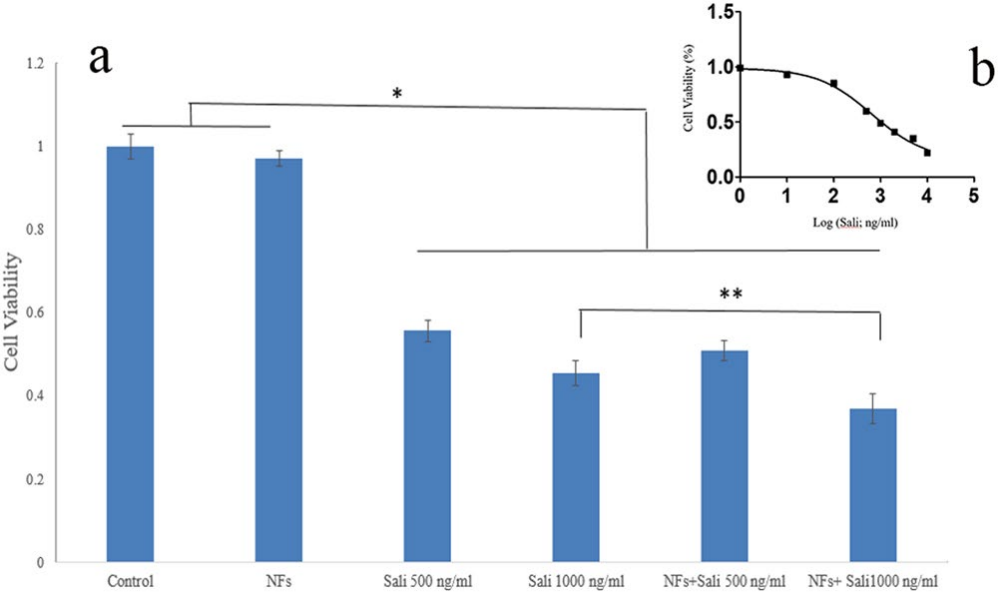
(**a**) Cytotoxicity of Sali and NFs + Sali on U251 cells after 48 h, using MTT assay. * indicates significant difference at p < 0.05. (**b**) Logarithmic graph of Sali IC_50_ on U251 cells. Data was presented as mean ± S.D, and n = 3.

The cytotoxic effects of Sali were examined in U251 cells, an established GB cell line. Exposure of U251 cells to Sali produced a concentration-dependent effect on cell viability with an IC50 value of 620 ± 70 ng/ml (Fig. 4). Based on these MTT viability studies, two different concentrations of Sali were chosen to allow a more focused look at activity of the NFs with concentrations of Sali expected to produce half-maximal inhibition of cell viability. Viability studies at the 500 ng/ml concentration of Sali showed a slight increase in cytotoxic responses in the PLGA + Sali nanofibres compared to free Sali (Fig. 4). This trend was even greater at the 1000 ng/ml concentration with Sali and NFs + Sali (1000 ng/ml) treatments resulting in cell viability of 49 ± 4% and 39 ± 3%, respectively. The PLGA NFs alone did not show any cytotoxicity in U251 cells. These studies suggest that Sali is an effective drug to induce cytotoxicity in human glioblastoma cells and stop their proliferation. In addition, NFs releasing Sali were found to be more effective than free Sali to induce cytotoxicity in U251. This may be attributed to the gradual release of Sali from the nanofibers over the 48-hour treatment period.

**Figure 5:**
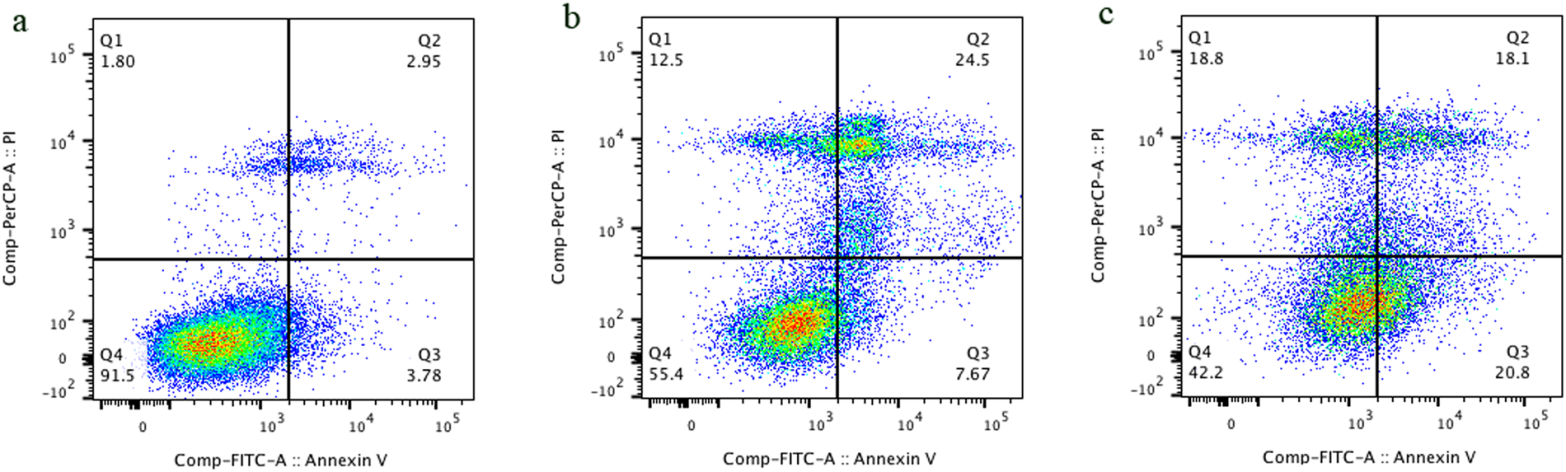
Cell apoptosis/necrosis of U251 after 48 h of the treatment, stained with Annexin V-FITC and PI for flow cytometry. (**a**) Control, (**b**) Sali and (**c**) NFs + Sali treated cells. (Q4) Live, (Q3) early apoptotic, (Q2) late apoptotic and (Q1) necrotic cells.

**Figure 6:**
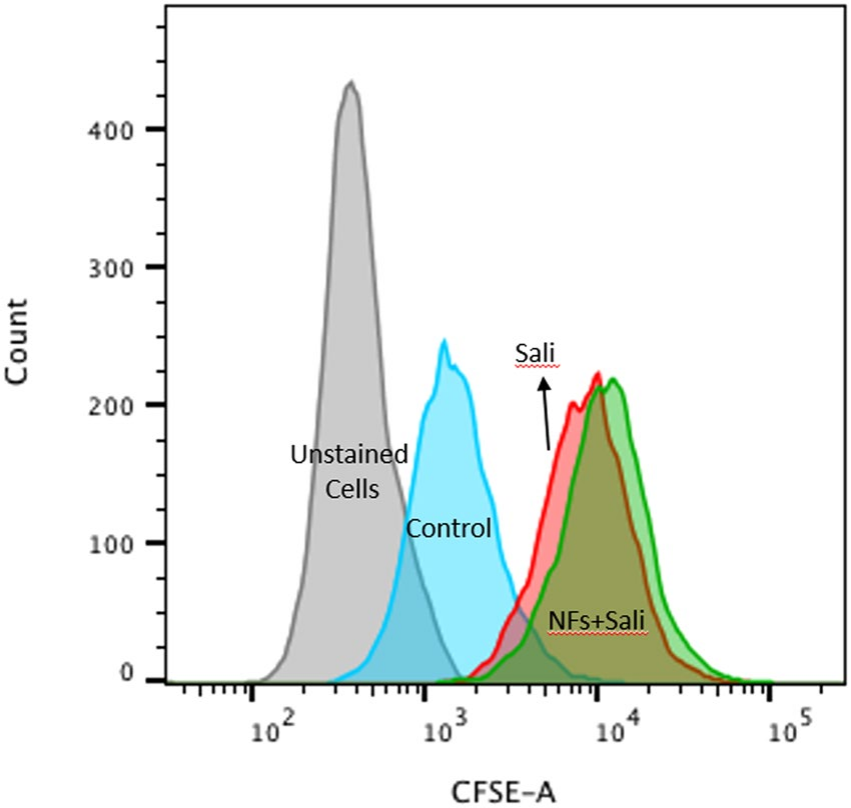
Cell proliferation analysis of CFSE-labelled U-251 treated with Sali and NFs + Sali after 48 h of the treatment.

**Figure 7:**
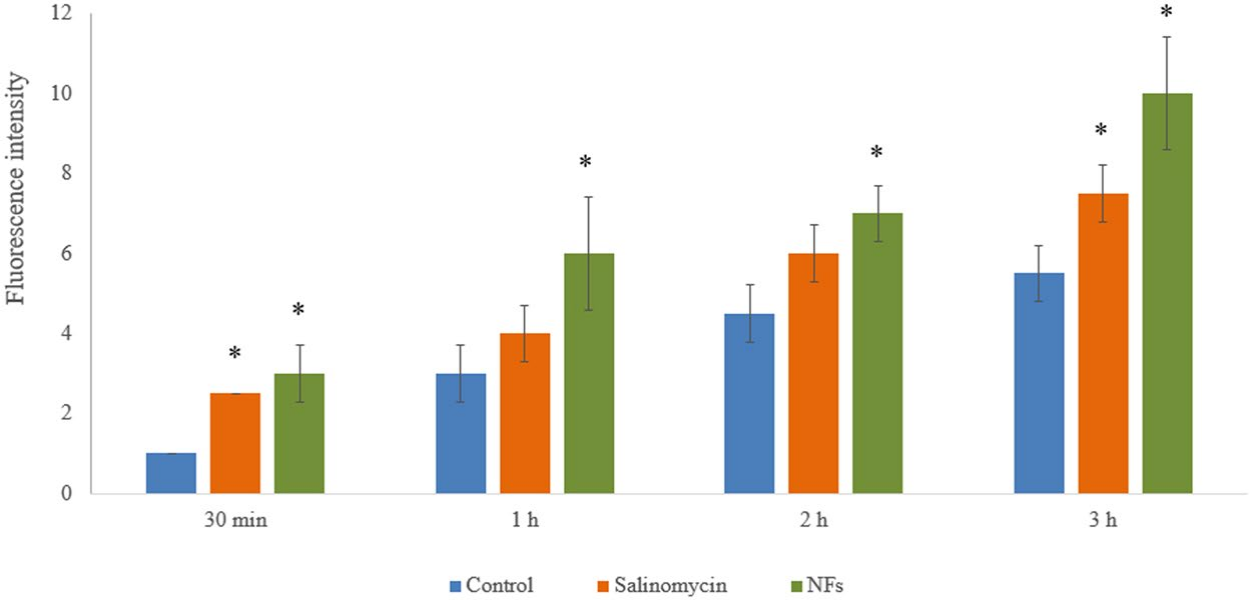
ROS concentration in U251 treated with Sali and NFs + Sali at different time-points. * indicates significant compared to the control group at p < 0.05. Data was presented as mean ± S.D, and n = 3.

To confirm the effects of Sali and nanofibres on U251 cell viability, flow cytometry-based analysis was performed (Fig. 5). The flow cytometry studies showed that both Sali and NFs + Sali treatments were effective at inducing apoptosis and necrosis in treated U251 cells, and reduced cell viability to 55.4% and 42.2%, respectively. As observed in the MTT studies, the flow cytometry analysis also indicated that NFs + Sali was more effective than Sali (p < 0.05) at inhibiting cell proliferation (Fig. 6). The anti-proliferative and cytotoxicity of Sali in the U251 cells has been reported previously in several different types of cancer cells such as leukemia^36^, prostate^37^, colon^38^ and ovarian cancer cells^39^. While the cytotoxic mechanism(s) for Sali are unknown, Sali-mediated ROS generation is known to be an important event committing the cancer cells to apoptotic death^37^. Indeed, ROS-mediated DNA damage and induction of G1 cell cycle arrest was suggested as the mechanism responsible for the increased effectiveness of Sali in U251 cancer cells compared to other agents such as cis-platinum^40^. In the present study, the generation of ROS in U251 treated with Sali and NFs + Sali was studied at various time-points (Fig. 7). As it can be seen, Sali, in both solution and nanofiber formulations, could effectively increase ROS production in a time-dependent manner. This is consistent with the study done by Xipell *et al*.^41^, reporting salinomycin can trigger ROS generation in glioblastoma cells in a concentration-dependent manner.

**Figure 8:**
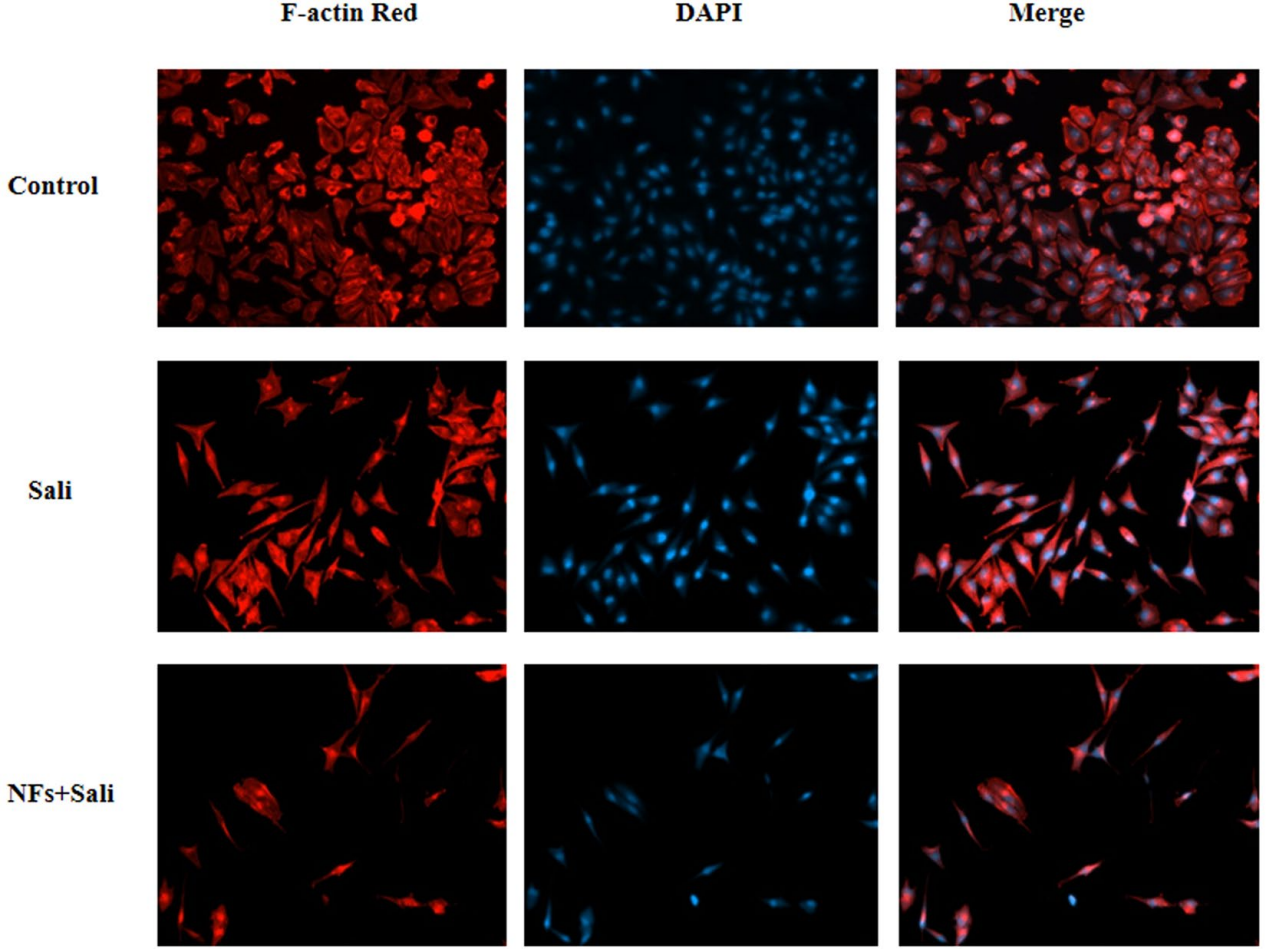
Fluorescence microscopy images of U251 treated with Sali and NFs + Sali after 48 h of the treatment. Red and blue fluorescence represents Alexa Fluor@ 488 phalloidin-stained F-actin and DAPI-stained cell nuclei, respectively.

The cell morphologies were also studied by fluorescence microscopy as shown in Fig. 8. In addition to decreasing cell proliferation, both Sali and NFs + Sali treatments produced morphological changes, including cellular shrinkage and cytoskeletal damage, that were consistent with apoptotis^42^ in U251 cells. Normal cells showed the typical cuboidal morphology of U251 while the treated cells depicted shrunken morphology with a spindle-like structure. The effect of salinomycin on changing morphology of other cancer cells and induction of mitochondria-dependent apoptosis has also been reported^39,43,44^.

**Figure 9:**
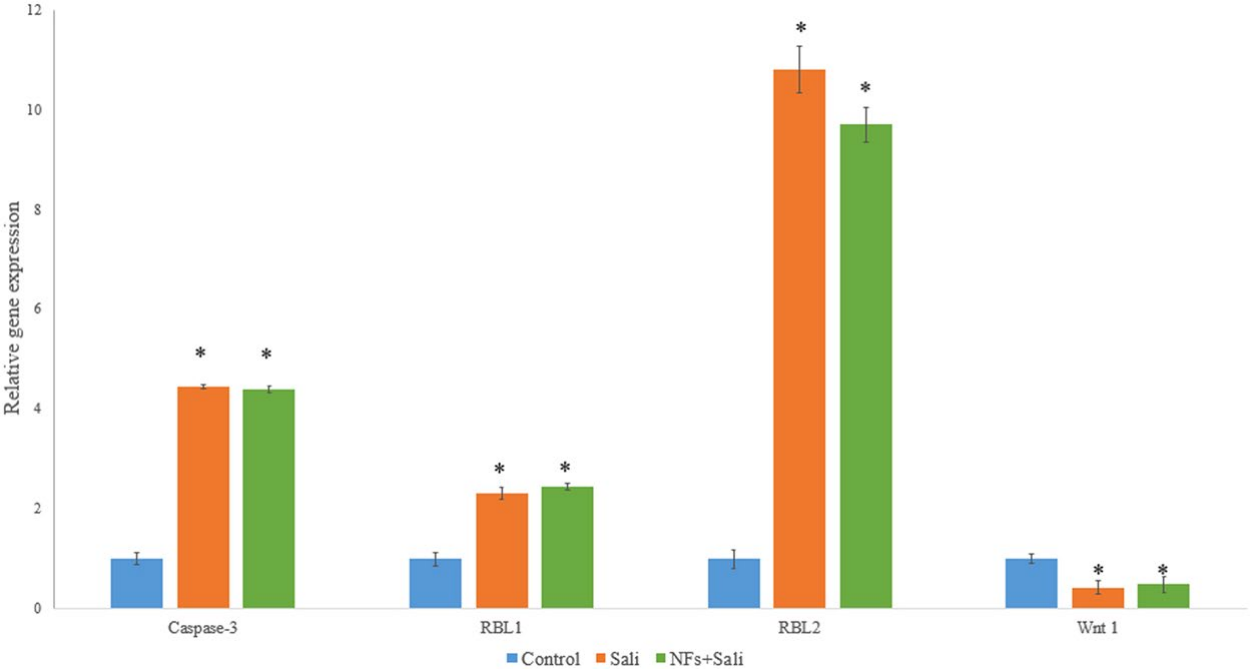
Relative gene expression of U251 cell treated with Sali and NFs + Sali after 48 h of the treatment. * indicates significance difference compared to the control group at p < 0.05. Data was presented as mean ± S.D, and n = 3.

To study the effect of salinomycin treatments on the gene expression of the cancer cells, RT-PCR was conducted. Figure 9 demonstrates gene expression changes in the U251 cells treated with Sali and NFs + Sali compared to the untreated cells. As it can be seen, upon treatment, expression of Wnt1 decreased while expression of caspase 3, Rbl1 and Rbl2 increased.

Apoptosis is a programmed cell death process that necessitates a cascaded activation and execution of a series of regulatory molecules and cysteine-aspartic proteases, known as caspases. Stress agents such as ROS, ultraviolet radiation and anticancer drugs are considered as the apoptosis triggers. It has been reported that salinomycin can trigger caspase-dependent apoptosis by elevating the intracellular ROS level in prostate cancer cells^37^. Generally, caspases are triggered in a sequential manner with casp ase 12 activation triggering activation of e caspase 9 and the subsequent ‘effector’ caspase 3^45^. Treatment of U251 with either Sali or NF + Sali increased caspase 3 expression by 3-fold. Such effects on caspase 3 expression is consistent with a caspase-dependent apoptotic response to Sali, as has been reported in other studies (reference).There were other additional changes in gene expression observed in the U251 cells exposed to Sali in solution or nanofibers. The retinoblastoma (Rb) family (Rb-1, Rbl1, and Rbl2), are considered as the tumor suppressors which are dysregulated in a majority of human cancer cells. Rb can inhibit cell cycle progression by directly disabling the E2F family of cell cycle-promoting transcription factors^46^. We found that salinomycin can up-regulate expression of both Rbl1 (up to 2-fold) and Rbl2 (up to 10-fold) that can result in the inhibition of the cell cycle progression.

The Wnt signaling pathway regulating stem cell self-renewal can be involved in the pathogenesis of various types of cancer. It has been reported that salinomycin can inhibit Wnt signaling and induce apoptosis in lymphocytic leukemia cells^47^, breast cancer cells^48^ and gastric cancer stem cells^49^. Our results also reveal that treatment of U251 with either Sali or NFs + Sali could decrease Wnt1 expression. It has been reported that Wnt activation can promote neuronal differentiation of glioblastoma cancer cells and its signaling pathway plays a critical role in malignant transformation and tumor progression in gliomas^50,51^. In addition, Wnt silencing in glioma cells could reduce the capacity of intracranial tumor formation *in vivo*^50^.

## Conclusion

In this study, PLGA nanofibers containing salinomycin were fabricated by electrospinning technique. The NFs displayed time-dependent release ca. 80% of Sali in 4 days by a diffusion mechanism with remaining drug released from the NFs within 2 weeks upon initiation of nanofiber degradation. NF + Sali were found effective in killing U251 glioblastoma cells, with improved cytotoxicity compared to free Sali which was attributed to the gradual release of the drug over the 48-hour exposure period. Furthermore, both Sali and NFs + Sali were capable of inducing intracellular ROS, a determining factor committing the cancer cells to apoptotic death. Finally, gene expression studies revealed that NFs + Sali could upregulate expression of Rbl1 and Rbl2 tumor suppressor genes as well as caspase 3 that can lead to caspase-dependent apoptosis, and simultaneously decreased Wnt signaling pathway. Therefore, the NFs + Sali has the potential to be utilized as an implantable drug delivery system in the brain cavity after surgical resection of the tumor.

## Materials and Methods

### Materials

PLGA (Mw 35–45 kDa, LA:GA 50:50) was purchased from PolySciTech, USA. Salinomycin monosodium salt was purchased from Sigma Aldrich, USA. Dulbecco’s Modified Eagle Medium: Nutrient Mixture F-12 (DMEM/F12), fetal bovine serum (FBS), trypsin, penicillin and streptomycin were all obtained from Gibco, USA. ActinRed™ 555 was acquired from Invitrogen Life Technologies, USA. 3-(4,5-dimethylthiazol-2-yl)−2,5-diphenyltetrazoliumbromide (MTT), 4′,6-diamidino-2-phenylindole dihydrochloride (DAPI), 2′,7′-dichlorofluorescin diacetate (DCFDA), dichloromethane (DCM), dimethylformamide (DMF) were all purchased from Sigma Aldrich.

### Electrospinning

PLGA was dissolved in a mixture of DCM and DMF (4 :1, v/v) at concentration 18%(w/v) to prepare the electrospinning solution. While DCM is individually a suitable solvent for PLGA, the high dielectric constant of DMF can improve the electrospinning of PLGA. For the drug loaded-nanofibers, salinomycin with the concentration of 0.02%wt of the PLGA, was dissolved in DMF and then added into the PLGA polymer solution prior to the electrospinning and stirred thoroughly to form a homogeneous solution.

The electrospinning system (Inovenso NE300, Turkey) consists of several constituents including a power supply of high voltage, a polymer solution reservoir, a digital syringe pump and a conductive collection device. A positive voltage of 17 kV was applied to overcome the liquid surface tension of the polymer solution and enable the formation of polymer jet. The electrospun nanofibers were collected on a collector, placed 15 cm away from the syringe needle. The flow rate of the electrospinning solution was set at 1 mL/h and the experiment was administered at room temperature. The fabricated electrospun nanofibers were subsequently vacuum dried overnight to remove the residual solvents.

### Fiber morphology and characterization

The morphology of the nanofibers was observed by scanning electron microscopy (SEM, FEI, Nova NanoSEM 450) with an accelerating voltage of 15 kV after being sputter-coated with a 5 nm layer of gold–palladium alloy. Quantitative size determination was performed by recording the diameter of 100 randomly selected nanofibers as measured using Image-J analysis software (National Institute of Health, USA).

To measure the degradation rate of the NFs, 10 mg of the electrospun scaffolds were incubated in 2 ml PBS (pH 7.4) at 37 °C and placed on a rotating shaker platform (60 rpm). At predetermined times, samples were removed and rinsed with Milli-Q water, dried at room temperature and weighed. Finally, the sample weights were plotted against time to obtain the degradation profile of the electrospun nanofibers.

### *In vitro* drug release

The release kinetic of salinomycin from the nanofibers was assessed at 37 °C in PBS buffers at different pH levels to mimic the tumor environment (pH 6) and normal extracellular fluid or blood (pH 7.4). Samples of Sali-NF (10 mg) were placed in 2 ml microfuge tubes and were incubated in 1 ml PBS under shaking conditions (60 rpm). At various time points, the enter PBS buffer solution was collected and replaced with 1 ml fresh PBS. The concentration of salinomycin was measured using an Ionophore ELISA kit (Europroxima, The Netherlands), according to manufacturer’s instruction. The accumulative release of salinomycin from the nanofibers was calculated as a function of incubation time.

The drug release mechanism was studied by semi-empirical equation established by Siepmann and Peppas:

1 $$\frac{Mt}{M\infty }=k{t}^{n}$$

where the *M*_*t*_ and *M*_∞_ are drug cumulative release amount at time *t* and infinite time, respectively, *k* is a constant of drug release rate, and *n* is the release exponent, indicative of the mechanism of drug release. *n* = *0*.*45* indicates the Fickian diffusion mechanism, *ana* < *n* < 0.89 shows anomalous transport and *n* *=* *0*.*89* implies swelling-controlled release mechanism from cylindrical matrix^34,35^.

### *In vitro* cytotoxicity study

Authenticated human glioma cell line U251 (gift from Dr. Thomas Klonisch lab) was used for the cytotoxicity studies. The cells were cultured in DMEM/F12, supplemented with 10% fetal bovine serum and 1% penicillin-streptomycin.

The cytotoxicity of Sali-NFs against U251 cells was evaluated by the MTT assay. Briefly, the GB cells were seeded in 24-well plates at a density of 10000 cell/cm^2^ and incubated overnight at 37 °C to allow them to attach. Then, the medium was changed with a fresh medium (negative control) and the medium containing an equivalent amount of Sali corresponding to Sali released from NFs (positive control), the PLGA NFs and PLGA-Sali NFs for 48 h. Afterwards, the culture medium was removed and the cells were washed with PBS and incubated in medium containing 10% MTT reagent for 3 h at 37 °C. Then, the solution was removed and DMSO was added to each well to dissolve formazone crystals. Finally, the solutions were retrieved and the absorbance was measured by a microplate reader (Biotek, USA) at the wavelength of 570 nm. The relative cell viability was calculated by [OD]_test_/[OD]_control_ × 100%, and the average value was obtained from the five measurements.

Moreover, cell apoptosis was assessed using Annexin V-FITC/PI apoptosis Kit (Thermo Fisher Scientific, USA). For this purpose, the cells were treated similarly with Sali and PLGA-Sali NFs for 2 days, followed by staining with Annexin V-FITC and PI in compliance with the manufacturer’s protocol. Thereafter, the samples were analyzed using flow cytometry (BD FACSCanto II Flow Cytometer instrument (BD Bioscience)).

Additional studies to observe the effects of the treatments on the cell proliferation were performed using flow cytometry. For these studies, the cells were labeled with the fluorescent dye carboxyfluorescein succinimidyl ester (CFSE), 50 Mm for 20 min at 37 °C. Then, the cells were washed and treated with Sali and PLGA-Sali NFs for 2 days. Subsequently, the fluorescence intensity of the cells was measured using the flow cytometry.

The morphologies of the treated glioma cells were also studied using a fluorescence microscopy (Zeiss Axio observer Z1, Germany). For this purpose, U251 cells were washed with PBS and fixed with 4% paraformaldehyde for 20 min at room temperature, followed by rinsing with PBS. Then, the cells were permeabilized with 0.2% Triton X-100 for 5 min, and washed with PBS four times. Then, actin cytoskeleton was stained with ActinRed for 30 min followed by nucleus staining with DAPI solution (100 nM) for 5 min at 37 °C. Finally, the samples were washed with PBS and observed by the microscope.

### Reactive oxygen species (ROS) determination

Intracellular ROS was assessed based on the peroxide-dependent oxidation of the nonfluorescent 2′,7′-dichlorofluorescein diacetate (DCFDA) to the highly fluorescent 2′,7′-dichlorofluorescein (DCF)^52^. For these studies, cells were seeded in black 96 well plates at a density of 10000 cell/cm^2^ and cultured overnight. After washing the cells with PBS, DCFDA (20 µM) was added. Upon entering the cells, DCFDA was rapidly hydrolyzed to the cell impermeable DCF. Therefore, cells were incubated with for 45 min at 37 °C, after which the solution was removed and the cells washed and treated with Sali and NFs in PBS for 1 to 3 h. The accumulation of ROS in the cells in response to Sali and NFs were determined by measuring the oxidation of DCFDA to the fluorescent DCF using a Synergy HT fluorescent plate reader at Ex/Em = 485/535 nm.

### Quantitative RT-PCR

The U251 cells were treated with free Sali and NFs + Sali for 48 hours. Then, the cells were washed with PBS and their total RNA was extracted using TRIZOL reagent (Invitrogen, USA) according to the manufacturer’s protocol. The purity and concentration of the extracted RNA was quantified by UV-VIS spectrophotometry (NanoDrop, Thermo Fisher Scientific Inc, USA).

Thereafter, the level of mRNA encoding caspase-3, Rbl1, Rbl2 and Wnt1were determined using quantitative reverse-transcript polymerase chain reaction (qRT-PCR). The RT-PCR was conducted using iTaq Universal SYBR Green Supermix kit (Bio-Rad, USA) and β-actin was employed as the housekeeping gene. The reaction was performed in an Applied Biosystems 7300 Real-Time PCR system with the following parameters: 1 cycle of 10 min at 50 °C for the reverse transcription reaction, 1 cycle of 1 min at 95 °C for polymerase activation, 40 cycles consisting of 15 sec at 95 °C for denaturation and 1 min at 60 °C for annealing. Relative gene fold changes were calculated by the comparative C_t_ method (2^−ΔΔCt^) and expression of the target genes was normalized to the β-actin. The sequences of the primers are listed in Table 2.

**Table 2:** Sequences of human primers.

	Forward	Reverse
β-actin	AATGCCAGGGTACATGGTGG	AGGAAGGAAGGCTGGAAGAGTG
RBL1	CCGGAAGCAGAGGAGGATTC	GGGCACATAATCGCATTGGC
RBL2	GGTTCCCACTGAGTGATTACTGT	AGAAGCCTCCTATGCTCACG
Caspase 3	CTCTGGTTTTCGGTGGGTGT	CGCTTCCATGTATGATCTTTGGTT
Wnt1	CAACAGCAGTGGCCGATGGTGG	CGGCCTGCCTCGTTGTTGTGAAG

### Statistical analysis

All quantitative results were obtained from triplicate samples and data were expressed as the mean ± standard deviation (SD). Statistical analysis was conducted using the one-way analysis of variance (one-way ANOVA) and a value of P < 0.05 was considered to be statistically significant.

### Data Availability

The datasets produced during and/or analysed during the current study can be available from the corresponding author on reasonable requests.
